# Evolution of the Corrosion Products around MnS Embedded in AISI 304 Stainless Steel in NaCl Solution

**DOI:** 10.3390/ma17164050

**Published:** 2024-08-15

**Authors:** Dan Li, Hongliang Hao, Zhichao Wang, Edwin Ernest Nyakilla

**Affiliations:** 1 Ordos Research Institute of Energy, Peking University, Ordos 017010, China; dthaohongliang@163.com (H.H.); wzc_0307@126.com (Z.W.); edwinernestnyakilla@gmail.com (E.E.N.); 2 School of Energy and Power Engineering, Northeast Electric Power University, Jilin 132012, China

**Keywords:** quasi-in-situ, sulfides (MnS), corrosion products, AFM, FIB

## Abstract

The characterization and evolution of corrosion products deposited on/around MnSs, a typical kind of inclusive particle embedded in AISI 304 stainless steel, was analyzed using a quasi-in-situ method in a 3.5 wt.% NaCl solution. On/around the MnS inclusion, a corrosion product layer with spinel Fe_3−x_Cr_x_O_4_ as the main component was formed, with a thickness of several hundred nanometers. Below the layer, there was a cavity layer in which part of the MnS remained, forming secondary pitting along the MnS/matrix boundary. The mechanism of corrosion product deposition and evolution accompanied by MnS dissolution, as well as the characteristics of the corrosion products, are discussed.

## 1. Introduction

Type AISI 304 stainless steel has been widely used in many fields as a necessary basic material, yet corrosion remains a big problem limiting its long-term service in harsh environments [1,2,3]. It is well known that inclusions are the initiation points of pitting corrosion, especially in chloride-containing environments [4,5,6]. Sulfide inclusions (MnSs) are always considered as the nucleation sites for initiating pitting corrosion [7,8,9]. It is believed that MnSs destroy the integrity and protection of the nanoscale passive film on the stainless steel surface, exposing the sulfide directly to chloride ions and thus causing pitting corrosion or even more severe localized corrosion [1,10].

Extensive studies have shown the importance of sulfides in the initiation of corrosion attacks on stainless steel in chloride-containing environments. S. E. Lott et al. [11] studied the corrosion behavior of 304 stainless steel and noted that chemical changes around MnSs are a key factor in pitting corrosion. D. E. Williams et al. [12] reported that when steel is exposed to an aqueous environment, a very thin, porous, metal-deficient polysulfide skin forms at the boundary between the matrix and inclusion, which can initiate pitting corrosion. Some researchers also suggested that the boundary region between sulfide and the matrix is susceptible to attack and preferential dissolution [2,13,14,15].

The earliest view of MnS-induced pitting corrosion of stainless steel was that the attack starts from the sulfide, which is less noble than the surrounding passive film-covered matrix, and then spreads to the active uncoated matrix below the sulfide [1]. Subsequently, many researchers confirmed this standpoint experimentally and explored more details of the MnS-induced corrosion process [14,16,17,18]. The Muto group did extensive work to investigate MnS/MnS-induced dissolution in stainless steel with different sulfur contents, and the associated corrosion mechanisms were further refined [19,20,21]. Based on the experimental results, the mechanism of MnS-induced corrosion has been proposed as follows [22,23,24]: (1) dissolution starts at the MnS/matrix boundary; (2) the synergistic effect of sulfur species released by MnS dissolution and chloride ions promote the dissolution of the MnS and the matrix, forming trenches along the boundary; and (3) trenches grow and evolve into metastable or even stable pitting. It should be noted that the appearance of trenches is considered a precursor to pitting and is a key step; the formation of trenches indicates that the active matrix is directly exposed to chloride ions and will be dissolved. At this point, it must be noted that corrosion products must be generated when the matrix undergoes dissolution. By modeling the propagation of a single pit in stainless steel, N. J. Laycock et al. [25] suggested that the local concentration of corrosion products affects the current density of passivation and noted that the presence of a porous cover around the pit affects repassivation and pit propagation. W. Sun [26] used a simulation model of corrosion product deposition with time and found that corrosion products affect the depth of corrosion pits. J.E. Castle and Ruoru Ke [15] suggested that pit covers are formed on MnSs, and the pit covers act as a barrier to protect the local electrolyte from dilution and maintain the dissolution of MnSs. It can be seen that the influence of corrosion products on pitting corrosion cannot be ignored. However, most of the previous studies were conducted under applied voltage/current conditions, which may affect the corrosion process, and the corrosion products may be difficult to deposit during rapid corrosion. There are few direct experimental observations on the evolution of corrosion products around MnSs. It is unclear what kind of corrosion products are deposited and whether the corrosion products are porous and accumulate with time, as inferred from W. Sun’s simulations.

Therefore, this paper focuses on the corrosion products deposited on/around MnSs during the dissolution process of stainless steel initiated by MnS inclusions under near-natural corrosion conditions, i.e., no voltage/current was applied during the study, which is one of our series of work on MnS-induced localized corrosion of stainless steel [27,28]. Specifically, the type, characteristics, and deposition process of corrosion products on/around the MnS were studied using quasi-in-situ methods through multiple microscopes, and the possible effects of corrosion products on the development of pitting corrosion was discussed.

## 2. Experimental

### 2.1. Material, Specimen Preparation and Solution

Commercial-type AISI 304 stainless steel, after re-sulfurization (sulfur addition), was used as the sample in this study, whose elemental composition is listed in Table 1. The raw material was commercial-type AISI 304 stainless steel (SS) produced by JiangShan Special Steel Machinery Factory Co., Ltd. (Taixing, China). The re-sulfurized (sulfur addition)-type AISI 304 stainless steel containing large-size MnSs that can be clearly distinguished by microscope was used as the test sample. In the re-sulfurized process, the raw bar was heated in a vacuum induction furnace at about 1500 °C to a molten state, and electrolytic manganese and FeS were added to the molten steel. Electrolytic Mn and FeS were added to the molten steel, which was condensed and molded into high sulfur and high manganese bars. Subsequently, the oxides on the surface of the formed bars were removed to reveal the metal surface, and the bars were forged and rolled successively to make plates with a width of about 10 cm and a thickness of about 1.5 cm.

The specimens were cut into 10 mm × 10 mm × 3 mm coupons along the rolling direction, grounded with sandpaper step by step to a 3000 mesh, and then polished with diamond polishing paste successively down to 0.5 μm. All experiments were conducted in a 3.5 wt.% NaCl solution (near neutral) prepared from analytically pure sodium chloride and deionized water.

### 2.2. Quasi-In-Situ Immersion Tests

In order to observe the evolution of the corrosion products, three periods of immersion were performed, namely 20 h, 40 h, and 60 h. An equal volume of fresh solution was used for each immersion test to prevent some possible contamination of the solution. After each immersion, the samples were gently washed with deionized water to avoid the unnecessary removal of the corrosion products after being taken out from the cell. Prior to the immersion tests, the approximate locations of MnS inclusions in the specimens were recorded with an optical microscope (OM, Olympus X53, Toyoko, Japan), and diamond notches were made around these inclusions with a microhardness tester (INNOVATEST, MAASTRICHT, The Netherlands, Vickers FALCON 300G2 hardness tester with a normal load of HV10 and a hold time of about 10 s), which can be easily identified with the microscope. Thus, inclusions of interest were re-examined after further etching and transferred between different characterization instruments. The marked MnS inclusions in the specimens were marked with MnS-numbers (number = 1, 2, 3…). In addition, a diluted pickling solution containing hydrochloric acid and hexamethylenetetramine was used to remove the corrosion products formed on the surface of some samples after 60 h of immersion. The specimens were soaked in the pickling solution for about 3 s, cleaned with ethanol and deionized water, and then blown dry with cold air.

### 2.3. Surface Analyses

A field emission scanning electron microscope (FE-SEM, Zeiss SIGMA 500, Oberkochen, Germany) was used in combination with an energy dispersive spectrometer (EDS) to obtain morphological and elemental information (mapping) on inclusions and associated corrosion products. All top surface images were obtained in the secondary electron mode (SE) with an accelerating voltage of 20 kV and a working distance of about 11 mm.

An atomic force microscope (AFM, Bruker Dimension Icon, Karlsruhe, Germany) was used to map the topography and the corresponding surface Volta potential distribution of the tested micro area containing the marked inclusion (the test area of about 20 μm × 20 μm). Volta potential is a characteristic property of a metal surface which is relevant to the electrochemical process [29]. In the Volta potential map, dark regions represent lower surface potentials, and light regions represent higher surface potentials. The Peak Force Kelvin probe force microscope (Peak Force-KPFM, Bruker, Karlsruhe, Germany) mode, which evolved from the tapping mode, is a Bruker proprietary mode. In this mode, by using a conductive probe (silicon tip, SCM-PIT, Bruker, Karlsruhe, Germany), high-resolution (512 × 512 lines) images can be obtained with low and optimized force between the probe and the sample without damaging the probe and sample surface.

Raman spectroscopy can be applied for the identification and characterization of non-metallic inclusions [30]. A laser confocal Raman microscope from WiTec (LCRM, WiTec Alpha 300+, Ulm, Germany) was applied to obtain characteristic Raman lines around MnS after immersion at different times. An objective lens equipped with a long working distance can be used to find these marked MnSs. In this study, Raman shift regions (wavenumbers) from 0 to 1000 cm^−1^ were tested with a laser wavelength of 532 nm, a power of about 1 mW, and an acquisition time of 6 min for each spectrum. Subsequently, the collected data were analyzed and plotted using built-in software (Project Five 5.1).

FE-SEM was combined with the Auriga Focus ion beam (FIB) from Zeiss to acquire the cross-sectional morphology of marked MnSs after 60 h of immersion. The FIB employs the sputtering phenomenon and uses a gallium ion beam to cut the materials [31]. The test area containing the marked MnSs was found using SEM. Then, the surface was milled with the gallium ion beam, and a thin protective platinum layer was sprayed prior to the milling process. The angle between the FIB and SEM was 54°, and the accelerating voltage was 30 kV.

## 3. Results

### 3.1. Inclusion Identification

**Figure 1:**
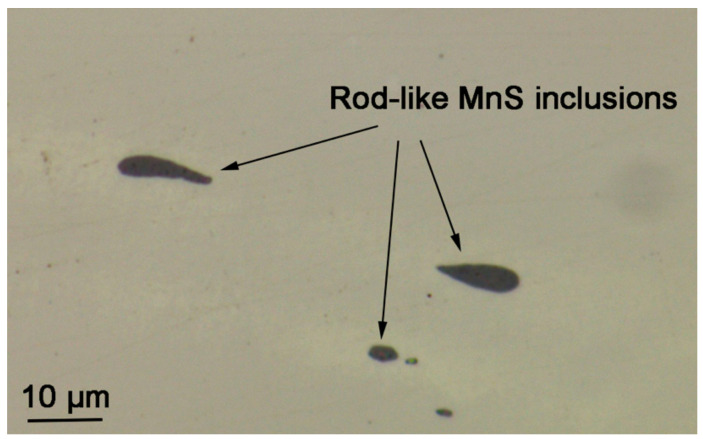
OM morphology of rod-shaped MnS inclusions formed in the re-sulfurized stainless steel.

**Figure 2:**
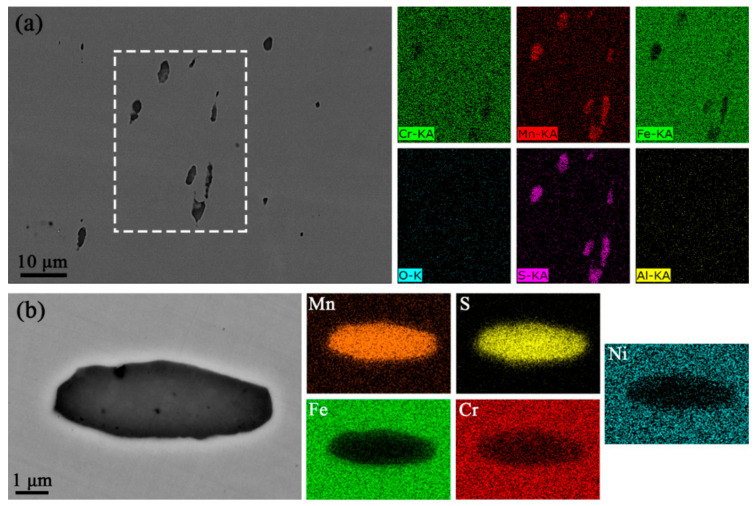
SEM morphology and EDS mapping results of (**a**) rod-shaped inclusions of different sizes in a wide field of view and (**b**) an individual inclusion.

As shown in Figure 1, the inclusions formed in the re-sulfurized stainless steel can be clearly distinguished using OM. After a large observation area of the specimens, it was found that inclusions in the specimens were rod-shaped and ranged in size from 3 to 12 μm. The EDS mapping results showed that the inclusions consisted mainly of elemental sulfur (S) and elemental manganese (Mn), i.e., sulfides (Figure 2). The atomic ratio of elemental Mn to S is 1:1, indicating that the rod-shaped sulfides are MnSs.

### 3.2. Quasi-In-Situ FE-SEM Observation of Corrosion Product Deposition

**Figure 3:**
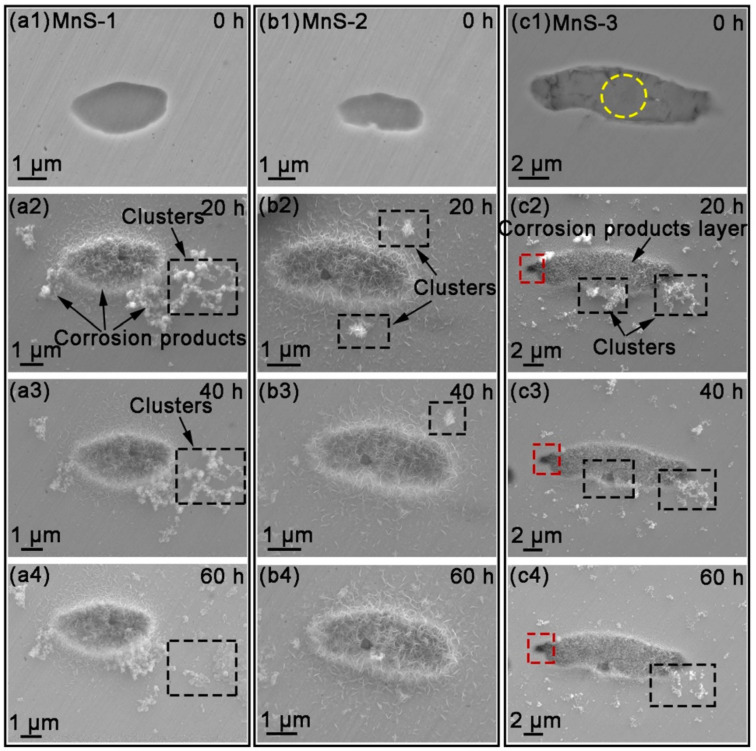
SEM morphologies of (**a**) MnS-1, (**b**) MnS-2, and (**c**) MnS-3 after immersion in 3.5 wt.% NaCl solution for 0 h, 20 h, 40 h, and 60 h, respectively.

**Figure 4:**
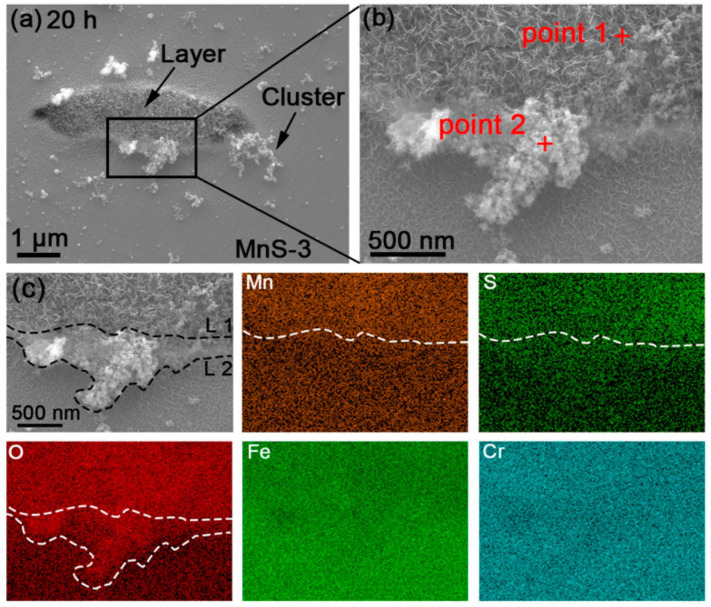
(**a**) Overall SEM morphology of the corrosion products deposited around MnS-3 after 20 h of immersion. (**b**) Enlarged image of the marked area in Figure 4a, and the corresponding (**c**) EDS mapping of the corrosion products.

It is widely believed that pitting corrosion starts at the boundary, where trenches are easily formed [4,32,33,34], and then the active matrix around the MnS is exposed to the solution and starts to dissolve. Figure 3 shows the morphology of MnS inclusions observed using FE-SEM after different immersion times (0/20/40/60 h). As previously described, the MnS inclusions were labeled prior to the experiments and labeled as MnS-1, MnS-2, and MnS-3, respectively (Figure 3(a1,b1,c1)). The corresponding EDS results for the three inclusions are shown in Figure S1. After 20 h of immersion, the corrosion product layer completely covered these inclusions, accompanied by some dendritic corrosion product clusters or scattered corrosion products randomly distributed on the surrounding matrix (Figure 3(a2,b2,c2)). These clusters appeared to be lightly attached to the matrix surface, and they may detach from the surface and partially disappear with increasing immersion time (Figure 3(a3,b3,c3,a4,b4,c4)), probably due to light rinsing in our quasi-in-situ tests. In contrast, the corrosion product layer appeared to be stable, as its top surface morphology did not change significantly with time. A magnified image of the corrosion products on/around MnS-3 at 20 h (Figure 4b) showed that both the corrosion product layer and clusters were composed of a large number of fin nanoscale needles.

### 3.3. EDS and Raman Detection of Corrosion Product Composition

EDS mappings were performed to obtain information about the distribution of elements in the corrosion products, which indicated that the main components of the corrosion products were elemental O, Fe, and Cr (Figure 4c). As a complement, the “film” (covering the upper region of the MnS) and “cluster” (as marked by red crosses in Figure 4b) were fixed-point analyzed, and their data are shown in Table S1, which were consistent with the EDS mapping results.

**Figure 5:**
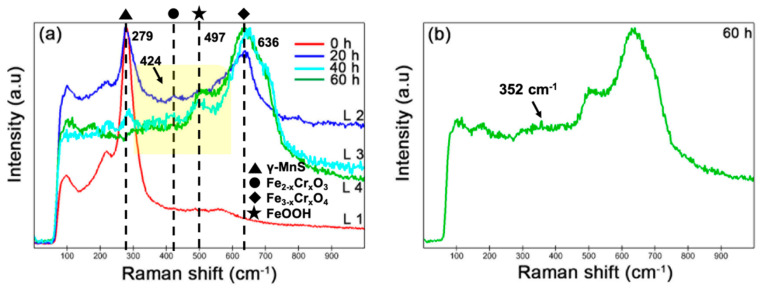
Raman spectra of MnS after different immersion times. (**a**) The peak at 279 cm^−1^ is MnS, whose intensity decreases with time; the peak at 636 cm^−1^ represents Fe_3−x_Cr_x_O_4_; the peak at 497 cm^−1^ appearing after 40 h of immersion belongs to FeOOH. In addition, the weak peak (424 cm^−1^) appearing at 20 h and 40 h may be Fe_2−x_Cr_x_O_3_. (**b**) An extremely weak peak at 352 cm^−1^ appearing at 60 h may be Cr_2_O_3_.

Since EDS can only provide us with an average signal of the thickness at the microscopic level, further Raman analysis was performed to gather more accurate information on the surface of the corrosion products. Figure 5 shows the Raman spectra of MnSs after different immersion times. The strong peak at 279 cm^−1^ before immersion (0 h) was the characteristic peak of γ-MnS [35]. After 20 h of immersion, in addition to the MnS peak, a new strong peak appeared at 636 cm^−1^ and a new weak peak at 424 cm^−1^, which are known to belong to the spinel structure Fe_3−x_Cr_x_O_4_ and corundum structure Fe_2−x_Cr_x_O_3_, respectively, according to the literature [36,37,38]. At 40 h, except for these three peaks that appeared at 20 h, a new peak appeared at 497 cm^−1^, which is the characteristic Raman line of FeOOH [39]. When the immersion time reached 60 h, only the Fe_3−x_Cr_x_O_4_ peak at 636 cm^−1^ and the FeOOH peak at 497 cm^−1^ were present. Meanwhile, an extremely weak peak at 352 cm^−1^ (Figure 5b) could be automatically identified by the matched software, and according to the literature, this peak may belong to Cr_2_O_3_ [40]. However, this peak was too weak to be considered as a normal Raman signal, and the peak will not be discussed in this paper. In addition, the peak at 424 cm^−1^, considered to be Fe_2−x_Cr_x_O_3_, which was present at 20 h and 40 h, was weak and difficult to avoid the suspicion of a background peak, so it is also ignored in this paper. It is noteworthy that the intensity of the MnS peak (279 cm^−1^) decreased with time, which was the opposite of that of Fe_3−x_Cr_x_O_4_, and eventually, the MnS peak disappeared after 60 h. Since the Raman detection area was located in the middle position of MnS-3 (the area enclosed by yellow dotted lines in Figure 3(c1)), the obtained Raman signal came entirely from the surface, i.e., the corrosion product film on MnS-3. Thus, it could be found that Fe_3−x_Cr_x_O_4_ was the main product of the corrosion product layer deposited on the MnS, and the slow increase in the peak intensity of Fe_3−x_Cr_x_O_4_ might imply that Fe_3−x_Cr_x_O_4_ was still being deposited on the MnS, or that the corrosion product film thickens with immersion time. Based on the SEM observations, the top surface of the corrosion product film hardly changed with time, and the FeOOH formed after 40 h of immersion could be the product formed underneath the Fe_3−x_Cr_x_O_4_. On the contrary, the decrease in the intensity of the MnS peak implies that the MnS was continuously consumed or that the thickening of the corrosion product film covered the MnS signal. The intervals of Raman spectral distributions of various substances reported in the literature are listed in Table 2.

### 3.4. AFM/SKPFM Measurement of Topography and Surface Volta Potential

**Figure 6:**
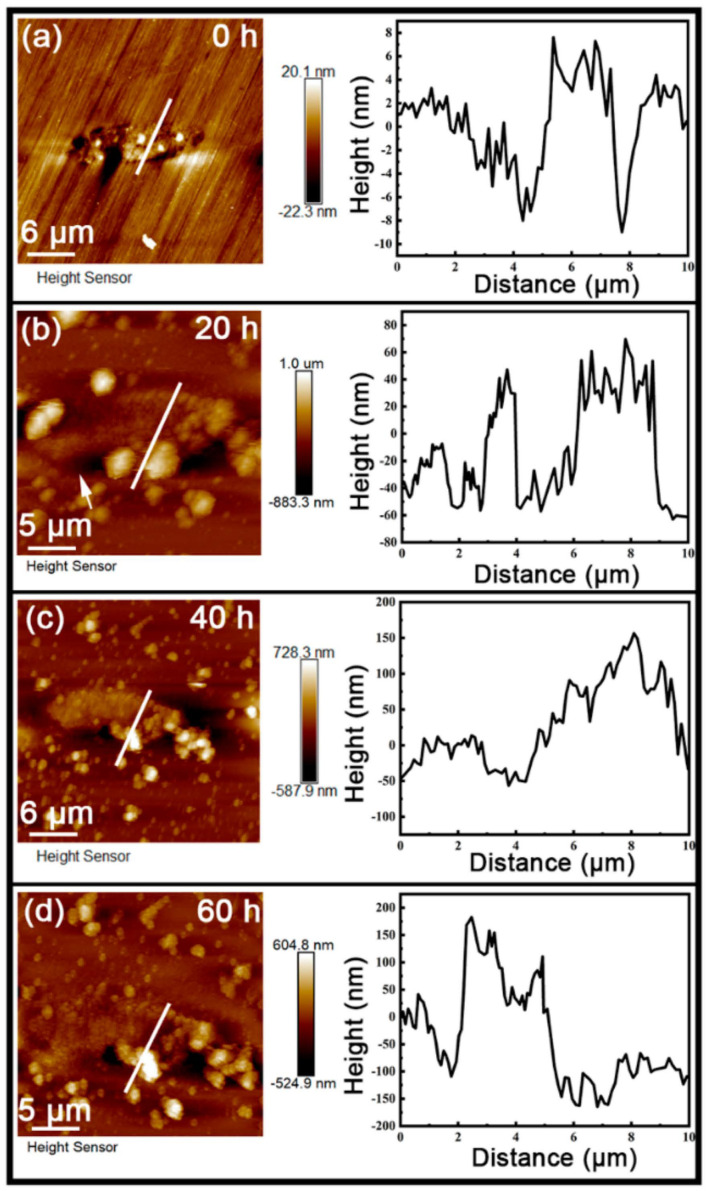
AFM topographies and correlation height profiles (white lines) of MnS-3 inclusion after immersion for different times: (**a**) 0 h, (**b**) 20 h, (**c**) 40 h, and (**d**) 60 h.

An AFM with a KPFM module was used to simultaneously examine the surface topography and the Volta potential distribution of the specimens. Figure 6 shows the topography of MnS-3 after different immersion times. As can be seen in Figure 6a, the height difference between the MnS and the surrounding matrix was within 20 nm at 0 h, then became approximately 100 nm at 20 h (Figure 6b), ~150 nm at 40 h (Figure 6c), and about 200 nm at 60 h (Figure 6d), which directly reflects the accumulation of corrosion products on the MnS over time, as we inferred from Raman spectra. In addition, the height difference between MnS-1 and the matrix and between MnS-2 and the matrix was also measured, and the line graphs are shown in Figure S2.

**Figure 7:**
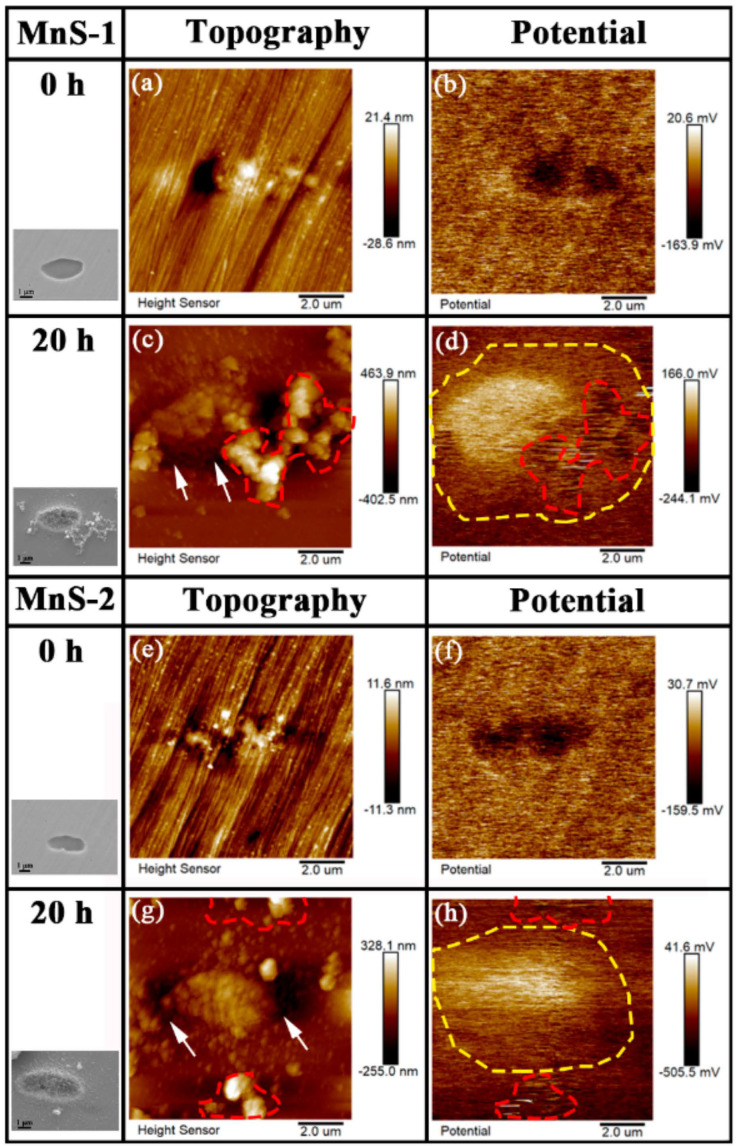
AFM results for MnS-1 and MnS-2. (**a**) Topography and (**b**) surface Volta potential of MnS-1 at 0 h; (**c**) Topography and (**d**) surface Volta potential of MnS-1 at 20 h; (**e**) Topography and (**f**) surface Volta potential of MnS-2 at 0 h; (**g**) Topography and (**h**) surface Volta potential of MnS-2 at 20 h.

Figure 7 shows the AFM topography and the corresponding surface Volta potential distribution for MnS-1 and MnS-2. The pristine MnS-1 (Figure 7a) showed a lower surface potential than the surrounding matrix before immersion (Figure 7b). However, the Volta potential of the corrosion product layer covering MnS-1 (Figure 7c) formed after 20 h of immersion was significantly higher than that of the surrounding matrix (Figure 7d). The same phenomenon was also observed for MnS-2 (Figure 7e–h). As can be seen from the potential map, the corrosion product layer covered a large area, including the MnS and the surrounding matrix (the area surrounded by the yellow dashed line), which was consistent with the SEM morphology in Figure 3c. As for those corrosion product clusters, whose surface Volta potential (area enclosed by the red dotted line in Figure 7d) was the lowest compared with the corrosion product layer and the surrounding matrix. As is known, the Volta potential refers to a potential difference between different electrodes, and each electrode has its own electrode potential. When two different electrodes (phases) are in contact, a potential difference is generated between them, and an electrolytic cell is formed between the two electrodes (phases) under the action of an applied voltage, which in turn results in a corrosion reaction. The potential difference also implies a possible driving force for the occurrence of galvanic corrosion, and the large the potential difference, the greater the tendency to corrode [1]. Therefore, the above Volta potential measurements suggest that the nobility decreases in the order of the corrosion product layer, matrix, and the corrosion product cluster. As mentioned before, the disappearance of these clusters may be due to the washing process, showing their unstable nature. Here, from the micro-galvanic corrosion point of view, those clusters with the lowest Volta potential are easily dissolved, also indicating the instability of the corrosion product clusters.

### 3.5. FIB-FESEM Observation of Cross-Sectional Morphology

**Figure 8:**
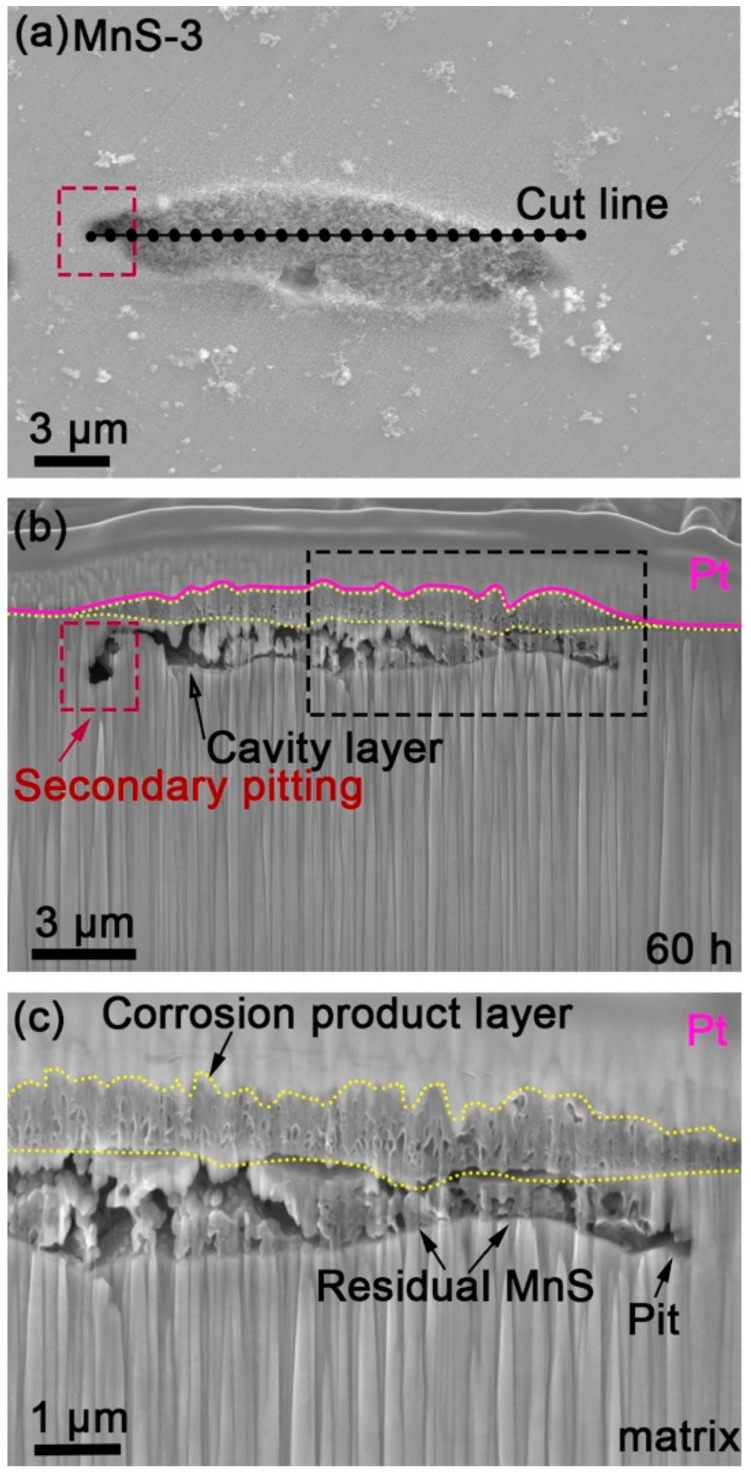
FIB-SEM images of MnS-3 after immersion for 60 h. (**a**) MnS-3 was cut along the cut line. (**b**) Cross-sectional morphology of MnS-3. (**c**) Magnified image of the area surrounded by black dashed lines in figure (**a**).

After analyzing the surface morphology of the corrosion products on the MnS inclusions, the cross-sectional morphology of the inclusion after 60 h of immersion was then analyzed using FIB-FESEM using MnS-3 as an example. The cross-section shown in Figure 8b was obtained by cutting along the cut line in Figure 8a. As be seen from Figure 8b, the layer (marked by yellow dashed lines) covering MnS-3 and the surrounding matrix under the platinum layer was exactly the corrosion product layer observed previously (Figure 3), with an overall gradual thinning from the MnS to the matrix. It can be seen that although the dissolution of MnSs leaves some voids in the pit under the corrosion product layer, the layer did not collapse in the pit, forming a locally closed environment. A “cavity layer” was formed beneath the residual MnS-3 inclusion, and secondary pitting was found along the boundary area between the MnS and the surrounding matrix, with a tendency to propagate toward the inner matrix (vertical direction). Figure 8c is an enlarged view of the area enclosed by black dashed lines in Figure 8b, which shows that the corrosion product layer is somewhat a porous layer, and its surface is not smooth and flat, which means that there may be some weak locations on the layer where the breakage of the corrosion product layer is likely to occur. In addition, the corresponding EDS line scan shows, to some extent, the elemental changes along the red line in Figure S3. It can be noted that the elemental O and Cr were more prominent in the intermediate position between the corrosion product layer and the residual MnS-3, probably Cr_2_O_3_. However, the EDS line scan provided a general trend of elemental changes in the local area for reference purposes only.

### 3.6. FE-SEM Morphology after Removal of Corrosion Product

**Figure 9:**
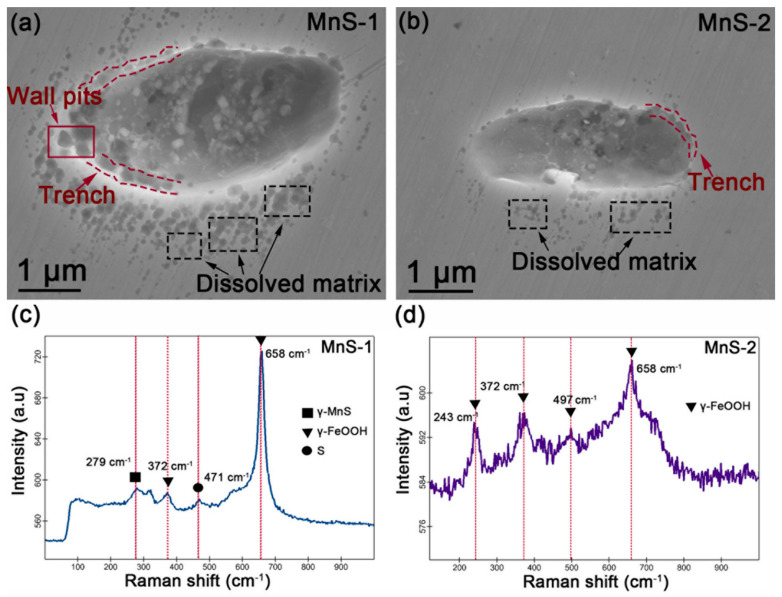
SEM morphology of (**a**) MnS-1 and (**b**) MnS-2 after removal of corrosion products formed after 60 h of immersion. Raman spectra of (**a**) MnS-1 and (**b**) MnS-2 collected in the area enclosed by the red circle. (**c**,**d**) Raman spectra taken at the middle of the pit formed by MnS-1 and MnS-2.

The AFM/SKPFM, FE-SEM/FIB-SEM, and Raman analyses described above depict the characteristics of the corrosion product layer deposited in the MnS region. The following pickling treatment was performed to remove the corrosion product layer and reveal the features under the layer. MnS-1 and MnS-2 were used as examples for the pickling treatment, and the corresponding FE-SEM morphologies are shown in Figure 9a,b. It can be seen that both the inclusions dissolved and corrosion pits were formed. Due to the limitation of SEM-EDS resolution, the EDS mapping in Figure S4 only shows the clear distribution of elemental O in MnS areas. The corresponding Raman spectra collected at the locations (the area surrounded by red circles) in Figure 9a and Figure 9b are given in Figure 9c and Figure 9d, respectively. The Raman signals were collected immediately after the removal of corrosion products. The morphologies in Figure 8a,b show the formation of “wall pits” as well as trenches along the MnS/matrix boundary, which are typical dissolution phenomena around the MnS. It is also noteworthy that many of the pits (the area surrounded by black dashed lines) were formed on the surrounding matrix that was once covered by the corrosion product layer, indicating that the passive film was disrupted in these areas, and the matrix was dissolved. In addition to these two inclusions, the same phenomenon was observed at other MnS inclusions, as shown in Figure S5. In Figure 9c, the peak at 279 cm^−1^ indicated that there was still residual MnSs in the pit of MnS-1, while the peaks at 658 cm^−1^ (strong peak) and 372 cm^−1^ (weak peak) were the Raman signals of γ-FeOOH. Additionally, the weak peak at 471 cm^−1^ in Figure 8c represents elemental sulfur, which is considered to be an important product of MnS dissolution and may be deposited in the pit; this phenomenon is consistent with other literature reports [41,42,43,44,45]. In Figure 9d, the peaks at 243, 372, 497, and 658 cm^−1^ are typical Raman lines of γ-FeOOH [39]. Thus, it can be inferred that γ-FeOOH exists in the pits beneath the corrosion product layer, whilst some MnSs and elemental sulfur may remain at the bottom of the pits. It must be mentioned that the pickling treatment may wash away some of the product in the pits, and some residuals can still be detected if one is lucky enough.

## 4. Discussion

**Figure 10:**
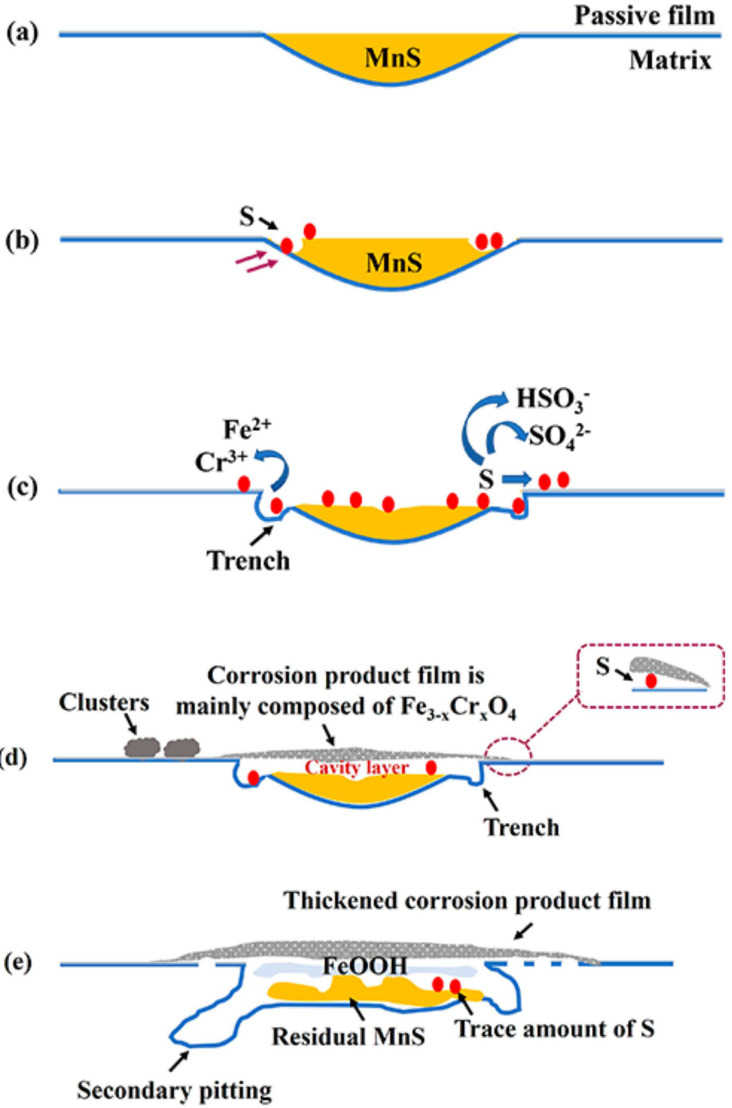
Brief schematic diagram of morphological changes on/around an MnS. (**a**) The original morphology of an MnS. (**b**) MnS dissolves from its boundary areas and releases S. (**c**) The peripheral active matrix is exposed and begins to dissolve, forming trenches along the MnS/matrix boundary. (**d**) As the corrosion product layer is deposited and covers the inclusion and its surrounding matrix, a cavity layer is formed under the layer. (**e**) The corrosion product layer slowly thickens with time, and in addition to the formation of FeOOH at the pit mouth beneath the layer, secondary pitting with a tendency to expand towards the inner matrix also occurs.

The corrosion products deposited on the MnS area were characterized and analyzed using the quasi-in-situ and ex-situ experiments described above. Accordingly, based on previous studies [28] and the experimental data obtained in this paper, the evolution of corrosion products accompanied by changes in the MnS morphology were discussed (see Figure 10 for a brief schematic representation).

From the surface, we observed two forms of corrosion products deposited on/around the MnS inclusions: layer and dendritic distributed clusters (Figure 3), both of which are composed of many nanoscale needles (Figure 4). The corrosion product layer thickens with the immersion time (Figure 6), and its thickness can reach several hundred nanometers. The Raman analysis shows that the main composition of the layer is Fe_3−x_Cr_x_O_4_ (Figure 5), and in addition, the layer has a higher surface Volta potential (Figure 7d,e) compared to the surrounding matrix (areas without corrosion product deposition), showing better stability to the external environment. In contrast, those corrosion product clusters distributed around the MnS inclusions have a lower surface Volta potential than the surrounding matrix and are slightly attached to the surface. These clusters have lower adhesion to the surface and, hence, easily fall off from the sample surface after a little washing. From the cross-sectional analysis, we found that an occluded zone was formed under the corrosion product layer, in which MnS was still present.

According to the results of SKPFM (Figure 7b,f), the surface Volta potential of the pristine MnS inclusion is lower than that of the surrounding matrix, so they act as small anodes and preferentially dissolve in the NaCl solution, while the passive film covering the surrounding matrix acts as a cathode [1,20]. MnS starts to dissolve from its boundary areas and directly releases elemental sulfur. Then, the surrounding active matrix is exposed and begins to dissolve, releasing Fe^2+^ and Cr^3+^ and forming trenches along the MnS/matrix boundary [22]. Sulfur, an important product of MnS dissolution, is directly released and deposited in the MnS region and then converted to other types of species (refer to reactions (1)–(3)). Those anions (SO_4_^2−^, Cl^−^) are concentrated in the trenches, creating a localized acidic environment, which in turn facilitates the dissolution of the MnS and peripheral active matrix [27].

MnS → S + Mn^2+^ + 2e (1)

S + 3H_2_O → HSO_3_^−^ + 5H^+^ + 4e^−^ (2)

HSO_3_^−^ + H_2_O → SO_4_^2−^ + 3H^+^ + 2e^−^ (3)

On the other hand, as can be seen from the cross-sectional morphology (Figure 9), the corrosion product layer is thick in the middle and thin on both sides, porous, and covering a large area involving the inclusion and the surrounding matrix. It has been reported that Cr forms oxides before Fe when the matrix starts to dissolve, as Cr oxides are thermodynamically easier to form than Fe oxides [45]. Moreover, the Cr_2_O_3_ formed at this time is not a dense and homogeneous passive film [46], when Fe ions pass through the Cr_2_O_3_ thin film to form Fe oxides, the Fe oxides and Cr oxides can react with oxygen to form Fe-Cr spinel oxides [37,47]. These spinel oxides are the main components of the corrosion product layer we observed in the current study, i.e., Fe_3−x_Cr_x_O_4_. It is noteworthy that the corrosion product layer composed of spinel Fe_3−x_Cr_x_O_4_ is porous without collapse, so it is speculated that the corrosion product layer may be a bilayer, and there may be a dense inner layer at the bottom that holds up the entire corrosion product layer.

Once a local environment is formed under the corrosion product layer, the local environment is different from that of the bulk solution. The corrosion product layer has a hindering effect on the transport and diffusion of substances. Therefore, chloride ions migrate to the cavity layer by electromigration and concentrate in the cavity layer together with those sulfur species released by MnS dissolution, forming a concentration cell in the cavity layer. Sulfur released by MnS dissolution is not only converted into sulfur species but is also partially presented in the form of elemental sulfur, as a weak Raman peak of sulfur can be detected in the pits after the removal of corrosion products (Figure 8c). Those elemental sulfur are not only deposited in the pit but also may be deposited elsewhere under the layer, such as on the matrix surface, due to the flow of liquid in the local area, which disrupts the passive film and dissolves the matrix into many small pits (Figure 8a,b). A similar point was made by Krawiec [48], who pointed out that elemental sulfur in MnS can react with the passive film on stainless steel, leading to the dissolution of the naked matrix. Moreover, the hydrolysis of metal ions can also lead to a decrease in the local pH, so the dissolution of the matrix and MnS still occurs in such a local environment, forming secondary pitting (Figure 9). During the hydrolysis of Fe ions, FeOOH is formed through the following variation process: Fe^2+^ + H_2_O → FeOH^+^ + H^+^ → 2Fe (OH)^2+^ + H_2_O → Fe (OH)_2_^+^ + H^+^ → FeOOH, and then remains in the pit mouth near the corrosion product layer [1]. Afterward, if it is fortunately retained after the washing process, it can be detected after the removal of the corrosion product layer.

**Figure 11:**
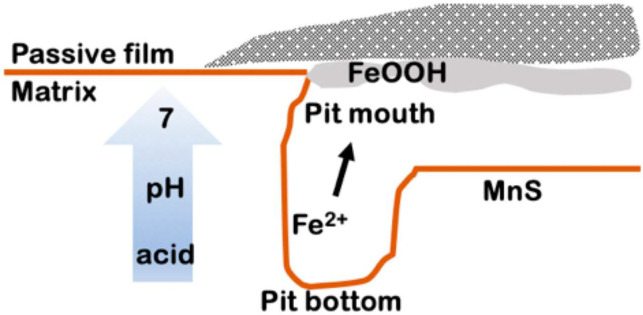
A brief schematic diagram of the trench along the MnS/matrix boundary.

Here, it is necessary to explain and discuss the formation of FeOOH. Although the precipitation of FeOOH occurs readily in alkaline environments, it may also occur in neutral or weakly acidic environments. Since the MnS dissolution and MnS-induced matrix dissolution in this study occur at natural corrosion conditions, which is driven by a micro-galvanic effect between the MnS and the matrix only, the entire dissolution process is slow. Under the corrosion product layer, although MnS dissolution and metal ion hydrolysis caused acidification of the local area, the acidity could only maintain the slow dissolution of the MnS and the active matrix, and there was a small pH difference in the pit. Thus, we believe that the pH near the pit mouth is close to neutral, where FeOOH can be retained (Figure 11 shows a brief schematic diagram of the trench along the MnS/matrix boundary).

The above discussion is only an analysis of the characteristics of the corrosion product layer formed on the MnS areas and the experimental phenomena that occur during its formation process. The deposition of corrosion products shows that the effect of the corrosion product layer on MnS-induced pitting corrosion cannot be neglected. The corrosion product layer acts as a barrier that separates the local environment around the MnS from the bulk solution, has an obstructive effect on the substance transport and migration, maintains the aggressiveness of the local environment, and eventually leads to the occurrence of secondary pitting.

To sum up, this work documents the deposition of corrosion products and analyzes the properties of the corrosion product layer. However, many factors influence localized corrosion, such as grain boundaries [49] and pH [50]. There are still some issues that need to be further explored and settled, such as speculation on the bilayer of the corrosion product layer. Based on the experimental results, a hypothesis during the deposition and evolution of corrosion products on/around MnS and the surrounding matrix was developed: the corrosion product layer has a dense Cr_2_O_3_ film on which the corrosion product layer, consisting of iron and chromium, accumulates over time, and that the existence of such a dense film ensures that the corrosion product layer does not collapse. Meanwhile, beneath the dense Cr_2_O_3_ film, FeOOH forms and exists in the pit formed by the dissolution of the MnS and the matrix. In future work, we will use more sophisticated instruments, such as high-resolution transmission electron microscopy (HRTEM) and 3-dimensional atomic probe (ATP), to explore the corrosion product layer and more details of the MnS-induced localized corrosion of stainless steel. In addition, a combination of simulation work and experimental work is considered to provide a comprehensive understanding of MnS-induced localized corrosion of stainless steel.

## 5. Summary

The characteristics and evolution of corrosion products deposited on/around the MnS, a typical inclusive particle embedded in AISI 304 stainless steel, were investigated and analyzed in this paper.

The MnS with lower surface Volta potential always preferentially dissolves in NaCl solutions and exposes the peripheral active matrix. When the matrix is dissolved, a corrosion product layer consisting mainly of Fe_3−x_Cr_x_O_4_ is formed on/around the MnS. The corrosion product layer covers a large area, including the MnS and the surrounding matrix. It has a significantly higher surface Volta potential than that of the surrounding matrix and thickens over time. The layer is porous, thick in the middle, and thin on both sides, and its thickness reaches approximately 600 nm. In addition, corrosion product clusters with a lower surface Volta potential are formed on the matrix around the MnS and remain for only a short period of time.

Under the corrosion product layer, part of the MnS remains, and a cavity layer is formed beneath the MnS. In addition, secondary pitting occurs along the MnS/matrix boundary and tends to expand toward the inner matrix.

## Figures and Tables

**Table 1:** Chemical composition of re-sulfurized type AISI 304 stainless steel.

Element	C	Si	Mn	P	S	Ni	Cr	Cu	Al	Fe
Re-sulfurized SS 304	0.073	0.72	2.81	0.022	0.043	8.17	18.1	0.24	0.013	balance

**Table 2:** The Raman spectra in the literature.

Substance	Raman Shift (cm^−1^)	Literature
MnS	Around 283 cm^−1^	[35]
Fe_3−x_Cr_x_O_4_	Between 542–531, around 636 cm^−1^	[37]
FeCr_2_O_4_	630 cm^−1^	[36]
Fe_2−x_Cr_x_O_3_	303, 424 cm^−1^	[37]
	Between 413–424, 515, 537 cm^−1^	[38]
FeOOH	243, 372, 497 and 658 cm^−1^	[39]
Cr_2_O_3_	353, 529, 553 cm^−1^	[37]
	353, 616 cm^−1^	[40]

## Data Availability

The raw data supporting the conclusions of this article will be made available by the authors on request.

## Supplementary Materials

The following supporting information can be downloaded at: https://www.mdpi.com/article/10.3390/ma17164050/s1. Figure S1. EDS results of (a) MnS-1, (b) MnS-2 and (c) MnS-3. Table S1. EDS spectra at the corrosion product film and cluster. Figure S2. Liner variation of height difference between MnS and matrix. Figure S3. FIB-SEM-EDS line scan results of the MnS-3 at 60 h (along the red line). Since all phases show almost the same color under the SEM, the different regions are divided into three parts along the red line segments: L1 represents the corrosion product layer, L2 represents the region of dissolved MnS, L3 represents the matrix underneath the MnS. Figure S4. SEM morphology of the inclusion labeled as MnS-A (a) before immersion and (b) after removal of the corrosion product film formed by immersion for 60 h. (c) Magnified image of the area surrounded by the black dashed lines in figure b. Figure S5. EDS mapping results of MnS-1 and MnS-2 after removal of the corrosion products.
