# Supplementary material for: Evolution of the Corrosion Products around MnS Embedded in AISI 304 Stainless Steel in NaCl Solution
Source: Materials (Basel). 2024 Aug 15;17(16):4050. doi: 10.3390/ma17164050 (PMC11356085; doi:10.3390/ma17164050)
Supplement: Supplementary file 1 [file materials-17-04050-s001.zip › materials-3126826-supplementary.pdf]

materials

MDPI

Supplementary

Evolution of the Corrosion Products around MnS Embedded in AISI 304 Stainless Steel in NaCl Solution

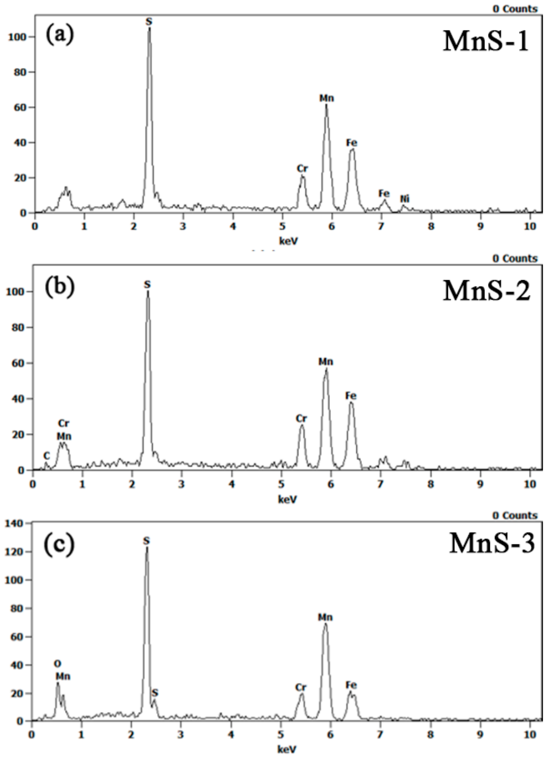

Figure S1. EDS results of (a) MnS-1, (b) MnS-2 and (c) MnS-3.

Table S1. EDS spectra at the corrosion product film and cluster.

Element (wt. %)	Fe	Cr	O	S	Mn	Ni
**Point 1**	58.21	16.70	14.30	1.83	5.41	3.55
**Point 2**	60.09	14.81	15.17	0.76	2.26	6.91

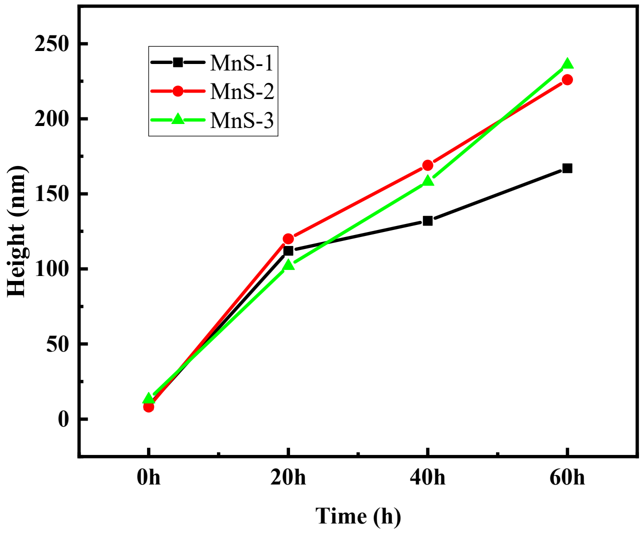

Figure S2. Liner variation of height difference between MnS and matrix.

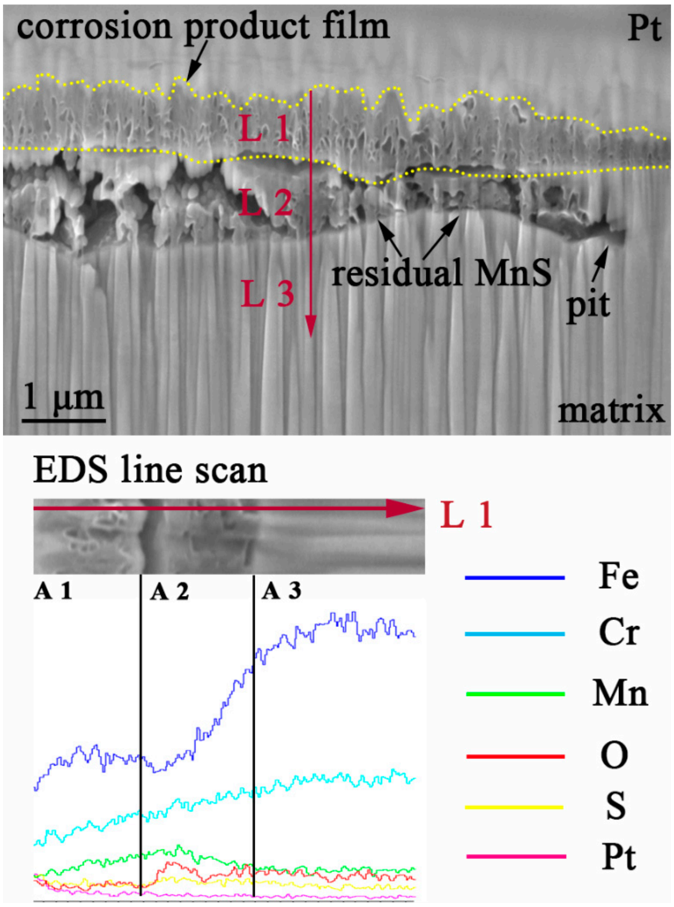

Figure S3. FIB-SEM-EDS line scan results of the MnS-3 at 60 h (along the red line). Since all phases show almost the same color under the SEM, the different regions are divided into three parts along the red line segments: L1 represents the corrosion product layer, L2 represents the region of dissolved MnS, L3 represents the matrix underneath the MnS.

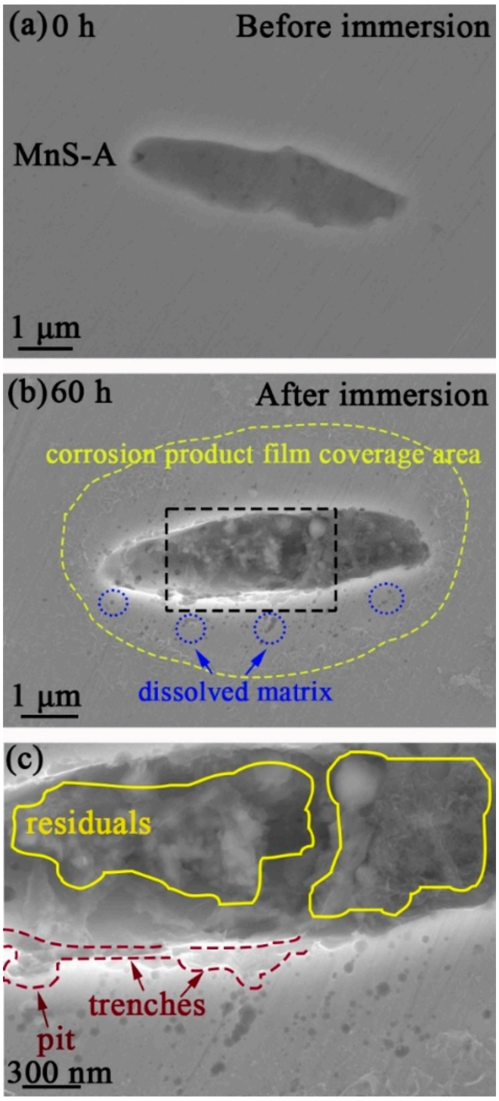

Figure S4. SEM morphology of the inclusion labeled as MnS-A (a) before immersion and (b) after removal of the corrosion product film formed by immersion for 60 h. (c) Magnified image of the area surrounded by the black dashed lines in figure b.

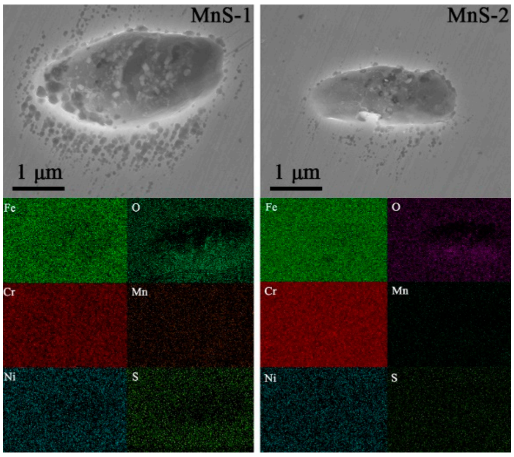

Figure S5. EDS mapping results of MnS-1 and MnS-2 after removal of the corrosion products.
